# Get In and Go: Why Health Care Needs the Patient at the Wheel

**DOI:** 10.1159/dib/acpag002

**Published:** 2026-10-10

**Authors:** Adam B. Cohen

**Affiliations:** a Global Health Mission Area, The Johns Hopkins University Applied Physics Lab, Laurel, MD, USA; b Department of Neurology, Yale Medicine, New Haven, CT, USA

**Keywords:** Artificial intelligence, Self-care, Digital health

## Abstract

**Background:**

US health care cannot meet demand. More than 100 million Americans lack basic primary care; shortages of over 80,000 physicians and 100,000 nurses are projected by the late 2030s; wait times keep rising; and roughly a quarter of national health spending is considered waste. Most digital health and artificial intelligence (AI) investment aims to make clinicians and health systems marginally more efficient, which cannot close a gap this large. Patients themselves can still manage only low-risk conditions, using over-the-counter products and consumer devices that gather data but rarely turn it into action.

**Summary:**

This article introduces Personal Health Care, a condition-agnostic and AI-enabled system architecture for what patients can do for themselves between clinical encounters, or instead of them, from prevention to treatment, and across the risk spectrum. Four conditions now make it feasible: system strain, generative AI that has reached clinical utility, patient-generated health data, and available capital. The architecture rests on five capabilities: sensing; deciding and acting; monitoring, governance, and safety; escalation; and external integration. A case of a poststroke patient with a possible breakthrough seizure shows the difference between silence between visits and continuous presence. The article also asks whether such systems should be measured against expert gold standards or against usual care, and introduces the series of articles, each taking up one capability.

**Key Messages:**

Provider-facing technology alone will not close the access and cost gap, so patients need capabilities they control. Personal Health Care is a system architecture rather than a single product, defined by five required capabilities. The limiting factors are validation, governance, liability, and reimbursement, not model capability.

“…To see takes time…”- *Georgia O'Keeffe*, New York, 1939[1]

## Introduction

### The Problem

In 1893, it was difficult to travel long distances. Getting from New York City to the Columbian Exposition in Chicago required access to a long-haul train [2, 3]. The Exposition was one of the greatest displays of innovation the world has ever seen [4]. One-third to one-quarter of the entire US population made the trip [4]. For the first time, there was the Ferris Wheel, moving walkways, fully electric kitchens and appliances, and industrial-scale electric power [4, 5]. Westinghouse, armed with Tesla’s patents, won the contract to electrify the entire fair, bringing it all to luminous life [5]. You’d have been there too.

Yet, there was a problem. A round-trip ticket cost roughly 500–900 USD in today’s dollars, and the journey took at least 20 h each way on the fastest premium service [3, 6] (Fig. 1). People could not just “go it alone.” That would have required excellent navigation and survival skills across harsh and varied environments, and the ability to operate, maintain, and repair horse-led caravans or carriages. In the 1890s, people relied on the skill and technologies of company-run rail conductors and operators. They could travel far, but most needed others to get them there.

**Fig. 1.**
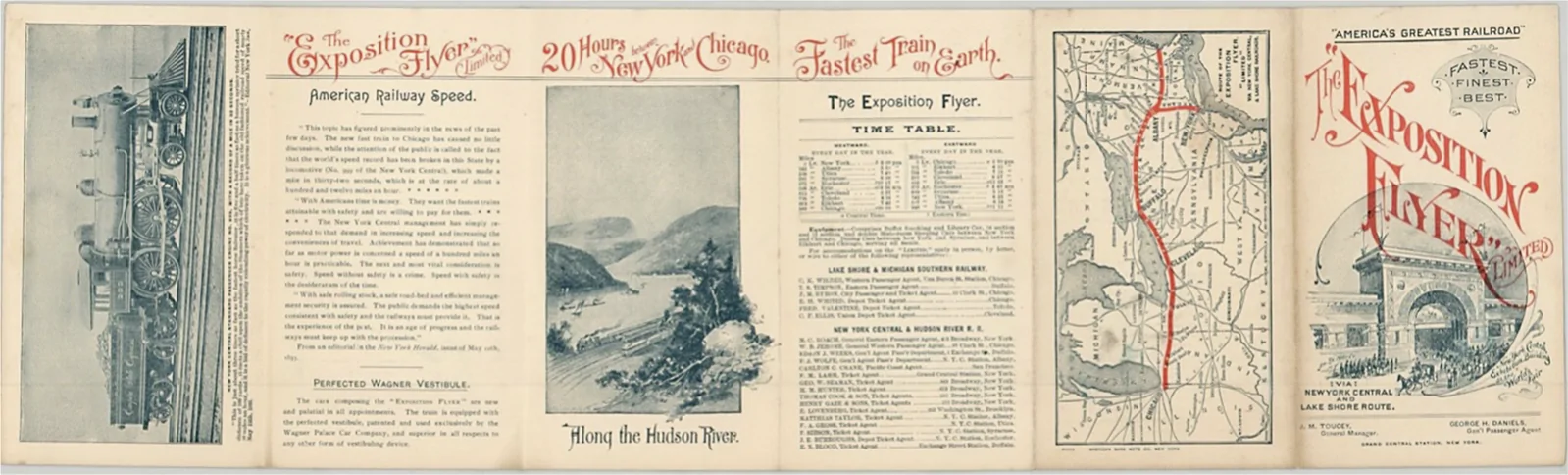
An 1893 advertisement of the New York Central railroad route to the Columbian Exposition in Chicago. [7].

Today, you can drive there yourself.

Get in your car. Enter your destination. Go.

To address most health needs today, patients can do very little themselves. They heavily depend on inaccessible, costly resources. Over 100 million Americans lack basic primary care, a problem that continues to worsen [8, 9]. An estimated shortage of over 80,000 physicians and 100,000 nurses will exist by the late 2030s [10, 11]. Wait times for new patient physician appointments average about 1 month and continue to rise [12]. While spending more than any other country on healthcare (at least 18% of the gross domestic product), an estimated 25% of the spend is waste [13, 14].

Consider Mary, aged 67 years, 1 month out from an ischemic stroke and now living with the heightened risk of recurrent stroke and poststroke epilepsy, an infrequent complication of ischemic stroke. She takes apixaban, atorvastatin, losartan, and levetiracetam. Last week, her husband noticed her blood pressure was out of range. She has yet to see her new neurologist, who is 90 min away, and her first appointment is 7 weeks out, well past the 34-day national median wait [15]. The neurologist is out of town this week and has no cross-coverage. This morning, she had a possible breakthrough seizure, and her family was on the phone with the local hospital’s nurse triage line, who told them to see her neurologist ASAP or go to the ER. Her primary care physician retired this year. Mary needs help today, but the traditional health system cannot deliver the right-sized resource. She requires something that does not yet exist.

To expand long-distance travel, trains were the start. Cars were the solution. In healthcare, provider-controlled hospitals and clinics are the start. Patient-controlled Personal Health Care must be part of the solution. Patients need to be able to drive, too.

Dramatically curbing healthcare costs and closing access gaps cannot occur by only giving care providers new technologies. The great technology revolution in healthcare, fueled by generative artificial intelligence (AI), will not simply better arm the traditional health system.

It will arm patients directly.

## The Opportunity

We still need trains, but cars have become indispensable. We need to better equip doctors, nurses, hospitals, and clinics, but Personal Health Care solutions will also become indispensable.

Here are four reasons why now is the time:

1 .Strain: Extreme care costs and access problems are worsening, and unlikely to dramatically change with provider-controlled add-ons.
2 .AI: Emergent technology, and especially generative AI, has already reached a point of obvious clinical utility. It continues to improve at incredible rates.
3. Data: People can increasingly gather meaningful data to monitor their own health. They can also use digital tools to better understand their health and expedite access to and improve communication with their clinical teams. The interplay between generative AI tools and patient-generated health data will be a key catalyst for this revolution.
4. Funding: There is tremendous current funding, excitement, activity, and deployment of AI-relevant technologies in healthcare [16]. Most of this, however, currently aims to make providers and traditional healthcare workers marginally more efficient and effective, like note scribing, note summarization, medical knowledge availability, medical ordering, patient messaging, and patient risk stratification. Some of these innovations and implementations will make a big difference, but they cannot close the gap. It’s just too big.

A precedent for Personal Health Care exists: Patient-controlled capabilities have long been available. Over-the-counter remedies and reference material have been around in the US since and before its formation.

John C. Gunn first published the highly popular “Gunn’s Domestic Medicine, or Poor Man’s Friend in the Hours of Affliction, Pain and Sickness” (Fig. 2) in 1830 in Knoxville, Tennessee [17]. By 1920, there had been over 200 editions [18]! It helped solve the access problem for frontier and rural families who lived far from any doctor [18]. Had a Columbian Exposition visitor stopped at a local pharmacy (Fig. 3) in 1893, she’d have encountered at least 1,000 over-the-counter official US Pharmacopoeia products [19]. Today, at any CVS (Fig. 4) or Walgreens, consumers find an endless array of over-the-counter products across 800 FDA-approved active ingredients for over 1,400 different therapeutic uses [20]. Further, consumers can choose from an endless array of smartphone apps, watches, bands, rings, patches, and other wearables, nearables, and hearables to collect and track their own health data.

**Fig. 2.**
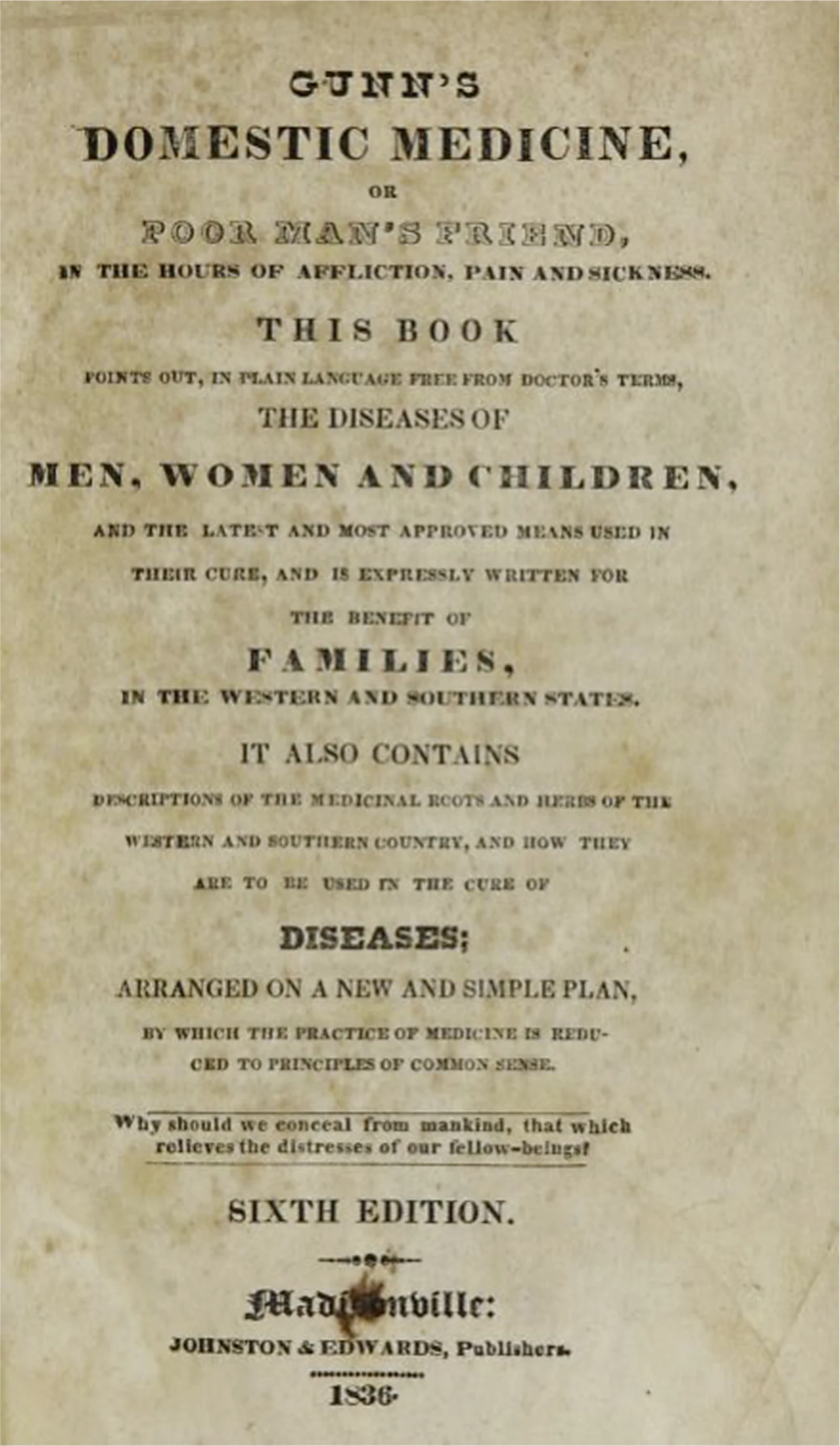
Gunn’s Domestic Medicine, or Poor man’s Friend in the Hours of Affliction, Pain and Sickness by John Gunn, 1830 [17].

**Fig. 3.**
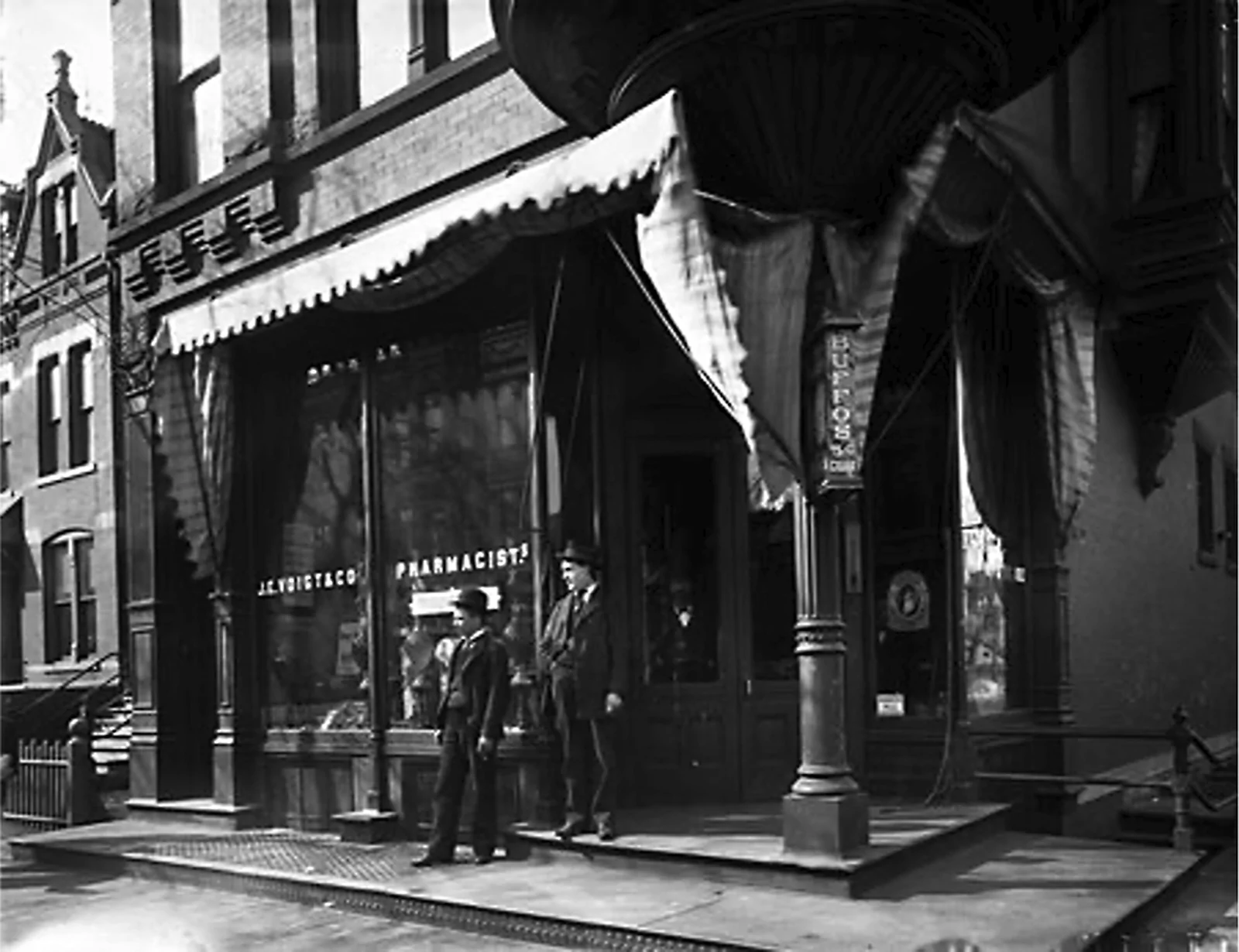
John E. Voigt and Benjamin Radke standing outside J.E. Voigt & Company pharmacy, located at 156 Center Street, Chicago, Illinois, Circa 1895. Center Street was later renamed Armitage Avenue [21].

**Fig. 4.**
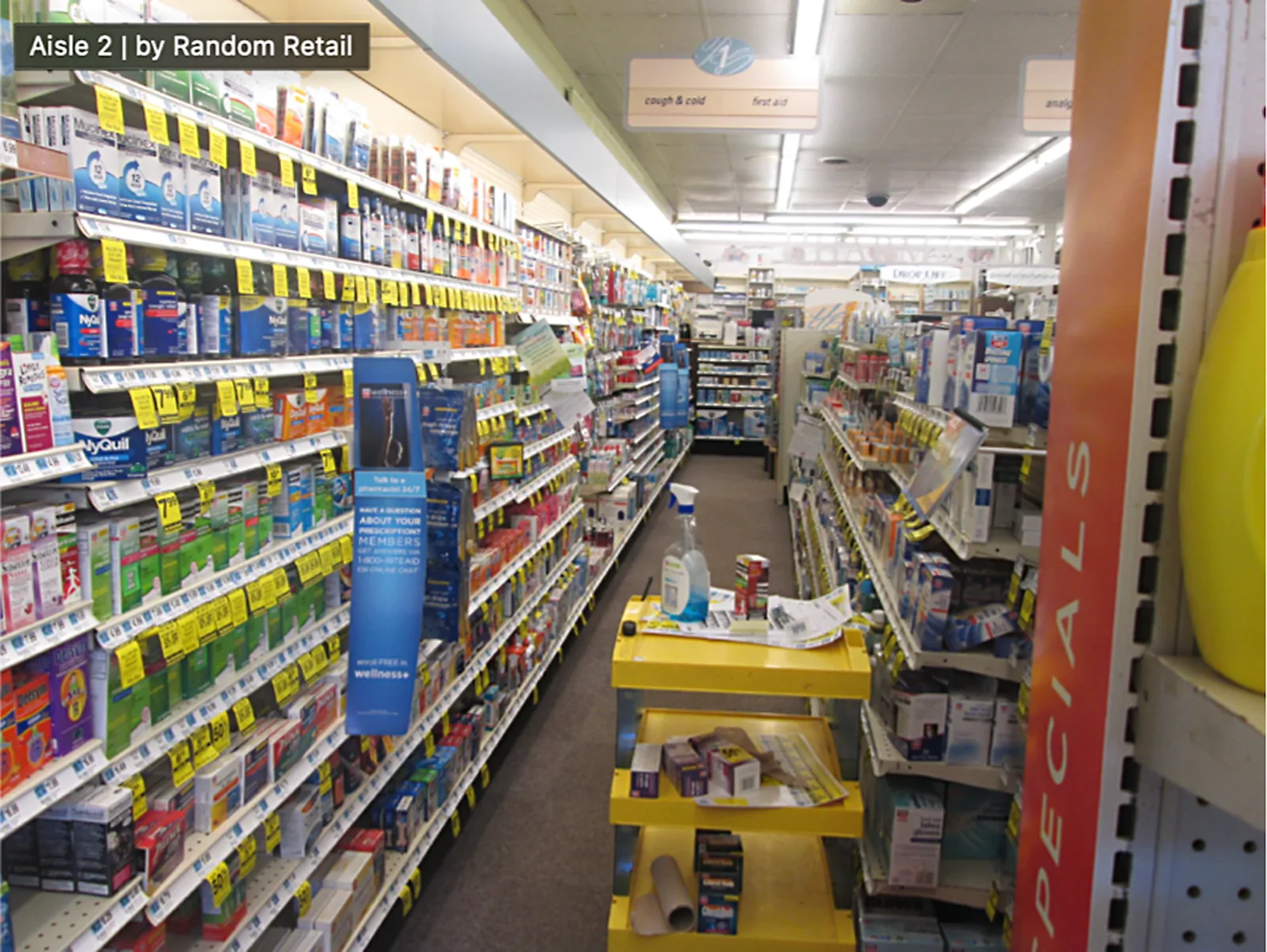
A view of the over-the-counter aisle at a current CVS. [22].

Yet, all these readily available pharmaceuticals and consumer technologies mainly address low-risk conditions like upper respiratory infections, headaches, and seasonal allergies. Further, data-gathering technology solutions largely lack the analyses and guidance support consumers need to turn data into action.

The WHO’s 2022 Self-Care Interventions guideline defines self-care as the ability of individuals, families, and communities to “promote health, prevent disease, maintain health and cope with illness and disability with or without the support of a health worker.” [23] It frames this as a global response to a projected shortage of 18 million health workers by 2030 and 400 million people without access to essential services. In practice, its recommendations narrowly focus on sexual and reproductive health and a slice of noncommunicable disease management. Agency for Healthcare Research and Quality’s (AHRQ) Self-Management Support framework is narrower still: a workflow model for primary care teams to support patients with chronic conditions [24]. Personal Health Care builds on both and goes further: It is a condition-agnostic AI-powered technology architecture across acute and chronic care, designed for use by patients across the *entire* risk spectrum and care pathway without requiring a clinician in the loop.

People need to get answers and take action now. They need to know what is going on with their body. They need to know what to do next. They also need to know the necessary actions to take, like getting tests and starting interventions. In many scenarios, waiting days or months for a test or treatment is OK. In other cases, waiting for an MRI or starting the medication is not OK. In other words, the traditional health system may say “wait,” but the pathophysiology will not comply. And beyond the concern of progressing pathology, waiting is suffering, too.

### Solution

Personal Health Care is not a single product or app. It is a system architecture for what patients can do for themselves between (or instead of) clinical encounters. Here is what that system must do:

There are two ways to optimize health. The first is to prevent health problems. The second is to deal with them when they occur. Personal Health Care addresses both. These two solution types may be controlled by patients, recommended by providers (and others in the traditional health system), or both. Today, the majority of “post-problem” tools are provider-controlled, while many prevention-focused tools are often only in the hands of the patient.

Physician, nurse, hospital, and clinic roles will evolve and will all remain essential, especially for high-acuity care, complex diagnosis, procedures, and the human dimensions of medicine, which patients will always seek. How that evolution plays out is uncertain and cannot be immediately determined. This includes the types and risks of tasks patients control versus those that require clinician oversight or at least shared-decision making. The extent of the shift itself will evolve as capabilities advance and safety and efficacy data mount. Much depends on how good Personal Health Care becomes, and on whether culture, regulation, and reimbursement adapt to make patient-controlled care viable for most patients.

Personal Health Care addresses six structural challenges (Table 1) of the current system that incremental fixes cannot address. Whether AI will replace doctors is the wrong frame. The real question is whether patients can get better access, equal or better quality, and lower cost than they get today, regardless of how it happens. For most patients, the assessment for a sore throat at 9 PM is a Google search, a ChatGPT session, or a nurse triage line. And for Mary, the poststroke patient with breakthrough seizures? It is a triage line that tells her to “see your doctor” for an appointment that is weeks away or head to the ER – there’s no middle ground. Personal Health Care must only be better than that.

**Table 1.** Six structural challenges of modern medicine that Personal Health Care (PHC) can address

Insight	Current state	What PHC enables?
Care happens in series; it could happen in parallel	Sequential assessments add weeks. Providers rarely coordinate timing	Concurrent input on the same case, with AI orchestrating the parallel workflows
No broad mechanism exists for providers to effectively deliberate with each other	Tumor boards exist for cancer; few other equivalents	AI-mediated multi-AI case “discussion,” accessible to any patient
Minimal outcome tracking, so we cannot learn what works	Outcomes return to providers as informal anecdote, rarely as structured data	Continuous structured outcome capture feeds back into both individual and population learning
No right-sizing of resources to pathway stage or complexity	Similar resources allocated whether a case is routine or complete and regardless of step of the pathway (e.g., triage, full diagnostic evaluation, monitoring)	Resource intensity scales with inference confidence and clinical risk
Learning must happen at both population and individual levels, each informing the other	Population evidence (RCTs and guidelines) and individual outcomes evolve in separate cycles	Each patient’s data sharpens the population model; the population model refines what the patient’s system recommends
We do not lack health data; we lack the right data in the right context and setting	Vital signs and activity dominate consumer data; clinically meaningful context is missing	Specialty-grade sensors deliver the right data, at the right moment, in the right setting

How effective is this baseline, the usual care policy?

It’s unknown.

In some scenarios, a Personal Health Care system or capability should match the gold standard a physician delivers. In others, it just needs to be better than no physician at all.

Fortunately, new technologies and systems will be held to a higher evidentiary bar than the unregulated and unmeasured triage and other clinical workflow paths patients already use under usual care. Yet, the bar is reachable, and demonstrations and attempts at validation are being published nearly weekly in top-tier journals. Though their study designs do not yet fully approximate real-world deployment, such methods are bringing rigor to quality and safety assessment of emerging Personal Health Care technologies. In controlled text-based consultations with simulated patients, Google’s AMIE matched or exceeded primary care physicians on most evaluation axes per specialist evaluators, with a multimodal follow-up extending the framework to image and laboratory reasoning [25, 26]. NEJM AI’s clinical reasoning benchmark, built on 750 script concordance items across specialties, offers an independent peer-reviewed measure on structured assessment [27]. These are not yet real-world deployments. They suggest the capability exists.

### Personal Health Care

Technology-enabled Personal Health Care is already underway. Examples in the market hint at what is possible. Direct-to-consumer prescribing platforms (Hims & Hers, Ro) deliver routine medications for sexual health, hair, and mental health without a clinic visit. Behavioral health apps (Headspace, Calm, BetterHelp) extend mental health care into the home. Chronic disease companies (Livongo, now part of Teladoc) couple connected devices with coaching for diabetes and hypertension. AI-enabled home diagnostics (Eko’s smart stethoscope, Tytocare’s home examination kit) let patients capture clinical-grade data outside a clinic. Continuous physiologic monitoring (Apple Watch ECG and AFib detection, Withings cardiovascular devices, Dexcom continuous glucose monitors) puts a low-grade clinical signal stream into millions of pockets. AI symptom-triage tools (Ada, K Health, Buoy) and emerging diagnostic-reasoning systems (Google AMIE, Microsoft MAI-DxO) point at the next layer of capability. Google’s Fitbit Air, launched in May 2026, and its Gemini-powered Google Health Coach (personalized fitness plans, injury-aware coaching) are a clear sign that consumer wearables are closing the sensing-to-guidance loop at scale [28]. Oura also rolled out Oura Advisor, an LLM-based coach built into the ring’s app, in March 2025 [29].

Each is a piece. None is a fully functional system, nor takes on the range of needs across varying clinical acuity, risk, and condition sets. For anything remotely high-risk, all still require a clinician somewhere in the loop to prescribe, validate, or interpret. Such tools extend the clinic’s reach more than they let the patient drive. The Personal Health Care architecture turns these pieces into a coherent patient-controlled system.

Dreaming up a system concept for Personal Health Care is easier than executing it. Yet, the system presented here would help patients care for themselves by preventing disease and addressing disease once it occurs, across an array of conditions for which they may currently completely depend upon a healthcare system that cannot fully serve them.

Engineers create development instructions in the form of requirements:

The machine shall dispense brewed coffee at a temperature between 160°F and 185°F.

The machine shall not permit coffee grounds to pass into the dispensed beverage.

At a conceptual level, the Personal Health Care system gathers all the information it needs to help you optimize your health, has the necessary decision-making capability to know what to do with that information, and then executes on those decisions at the right time.

The Personal Health Care system rests on five capabilities, each of which contains a set of requirements (Table 2). Sensing captures the right data, including history, physiological measures, and test results, at the right moments. Deciding and Acting reasons over that data with stated confidence and takes action within validated scope, which may include triggering the Sensing capability again. Patient context also shapes how much a patient can or wants to engage in Personal Health Care, influenced by general, digital, and health literacy, cognitive ability, and available resources. This layer must help route the patient to the right path, including not engaging at all: deferring to routine care or bringing in a helper, such as a family member. Monitoring, Governance, and Safety validates the system’s own performance, surfaces drift, learns at both population and individual levels, and stays within applicable laws and regulations. Escalation routes higher-risk scenarios, with full context, to traditional clinical care. External Integration connects everything to the records, prescribing, scheduling, and people of the existing health system. The role of clinicians and the system’s safety boundaries – what it can decide independently, what needs approval, and when it escalates – will sharpen as Personal Health Care is tested against benchmarks and deployed into the world. Those range from expert-level gold standards, where they exist, to usual care policy, where patients often face little or delayed guidance and access.

**Table 2.** Personal Health Care System capabilities and requirements

Capability	Requirements	Mary’s example	Why it matters?
Sensing	The system shall identify the clinical problem to sense, voiced by the patient or inferred from data The system shall access relevant data through validated sensors, devices, and patient/family input The system shall flag incomplete or unverified data before acting on it The system shall not act on data it cannot verify	Takes interactive history from Mary and her family. Detects the morning’s likely seizure via wearable EEG, accelerometer, electrodermal activity, and heart rhythm and rate data. Pulls home BP readings, smart pill organizer logs (apixaban, levetiracetam), gait and sleep data. Flags a 36-h EEG patch gap as data missing	Each sensor replaces a visit Mary cannot easily make. Bad or missing data is worse than no data, so the system must know what it does not know
Deciding and acting	The system shall assess the patient’s desires, literacy, cognitive ability, and resources, and route to the appropriate level of engagement, including deferral to routine care or a helper, when self-directed engagement isn’t appropriate The system shall reason transparently about confidence, evidence, and uncertainty The system shall act within its validated scope and defer to a clinician when actions exceed it The system shall not act when certainty falls below clinically meaningful thresholds The system shall not recommend or adjust therapies outside its validated scope	Correlates missed levetiracetam doses and seizure event. Separately notes BP trend as unrelated. Tells Mary, with stated confidence, that adherence likely drove the seizure and that recurrent stroke is not indicated. Adjusts medication reminders and recommends a losartan dose change but queues a home BP check and basic laboratories plus a virtual consult on the cause of the BP trend	Mary needs to know what to trust. Some dose changes require clinician judgment, and some may not. Speculative action causes harm
Monitoring, governance, and safety	The system shall continuously validate performance against intended use and population The system shall surface performance drops to users and operators The system shall learn at both population and individual levels, each informing the other The system shall comply with applicable data, device, and liability regulations The system shall not silently degrade The system shall not update safety-relevant components without re-validation	Tracks how often its seizure classifications match clinician interpretation over time. Tells Mary if reliability drops. Mary’s outcomes refine the population model and her self-model (what works for Mary?!); the population model also refines what Mary’s system recommends. Data is HIPAA-protected; algorithms are FDA-cleared where required; chain of liability is documented	Drift in patient state should not silently erode accuracy. Trust requires transparency, validation, and legal scaffolding. Both layers of learning improve only when connected
Escalation	The system shall recognize higher-risk scenarios and route them with full context attached The system shall escalate life-threatening events to emergency services with patient-relevant data pre-loaded The system shall not delay clinical handoff in time-sensitive scenarios The system shall not handle scenarios outside its validated scope	Status epilepticus triggers 911 with medication list, allergies, and recent monitoring data sent ahead. A cluster of seizures in 24 h auto-schedules an emergency room or urgent neurology visit with the seizure log and relevant health data attached	Seconds may matter. Intake delays worsen outcomes. Context-rich handoff improves what happens next
External integration	The system shall interoperate with health system records, prescribing, scheduling, and clinical and emergency services The system shall let patients share their data/record with new applications and clinicians on demand The system shall not silo patient data The system shall not bypass licensed prescribing or testing channels	Sends Mary’s seizure log and BP trend to her other providers before the appointment. Her full record is portable to any new provider she chooses. Pharmacy and laboratory orders route through licensed channels	Visits start with context, not intake. The patient owns the record. Personal Health Care cannot operate around the legal and clinical infrastructure that protects patients

For Mary, Personal Health Care does what the triage line cannot. Her wearables continuously monitor her brain, heart, and body. Her home cuff has logged a week of out-of-range blood pressure readings. Her seizure-detection suite of technologies classifies the morning event by body parts affected, severity, duration, and time. Her smart pill organizer flags two missed doses of levetiracetam in the preceding 48 h, and her sleep tracker hints at sleep deprivation, too. The system makes meaning of these signals and tells Mary, in plain language, that the breakthrough seizure was likely driven by missed doses, that her blood pressure trend needs attention soon, and that nothing in the data points to recurrent stroke or a more dangerous cause of seizure. It generates a structured event summary, modifies her home blood pressure regimen, executes a set of home laboratories, schedules a virtual visit check-in, and prepares a full briefing for her neurology appointment.

Personal Health Care works with her doctors and does what they cannot do. It compresses days of fragmented worry into hours of actionable information, and it brings the expertise of specialists to her when she needs it. It also brings specialists the right information when Mary actually sees them. For higher-stakes events like status epilepticus, signs of recurrent stroke, or hypertensive emergency, the system escalates to 911 or the nearest emergency department with Mary’s medication list, allergies, and recent monitoring data pre-loaded. An even better future is one with Personal Health Care safely and effectively handling the acute problem, avoiding the emergency department completely.

The default current state for Mary today is silence between visits. With Personal Health Care, the default state is presence: always there, sometimes quietly watching, already to help.

Mary’s story is one of many. The same gap holds for patients with progressive coronary artery disease, occupational chemical exposure, or any condition that demands more than episodic visits provide and less than emergency visits deliver. Personal Health Care is built for them, too.

A Personal Health Care system should also follow the “best of” what health systems and clinicians emulate (Fig. 5). Guiding tenets come from the Institute of Medicine’s Six Aims outlined in “Crossing the Quality Chasm” and The Accreditation Council for Graduate Medical Education’s (ACGME) Six Core Competencies for US graduate medical education [30, 31].

**Fig. 5.**
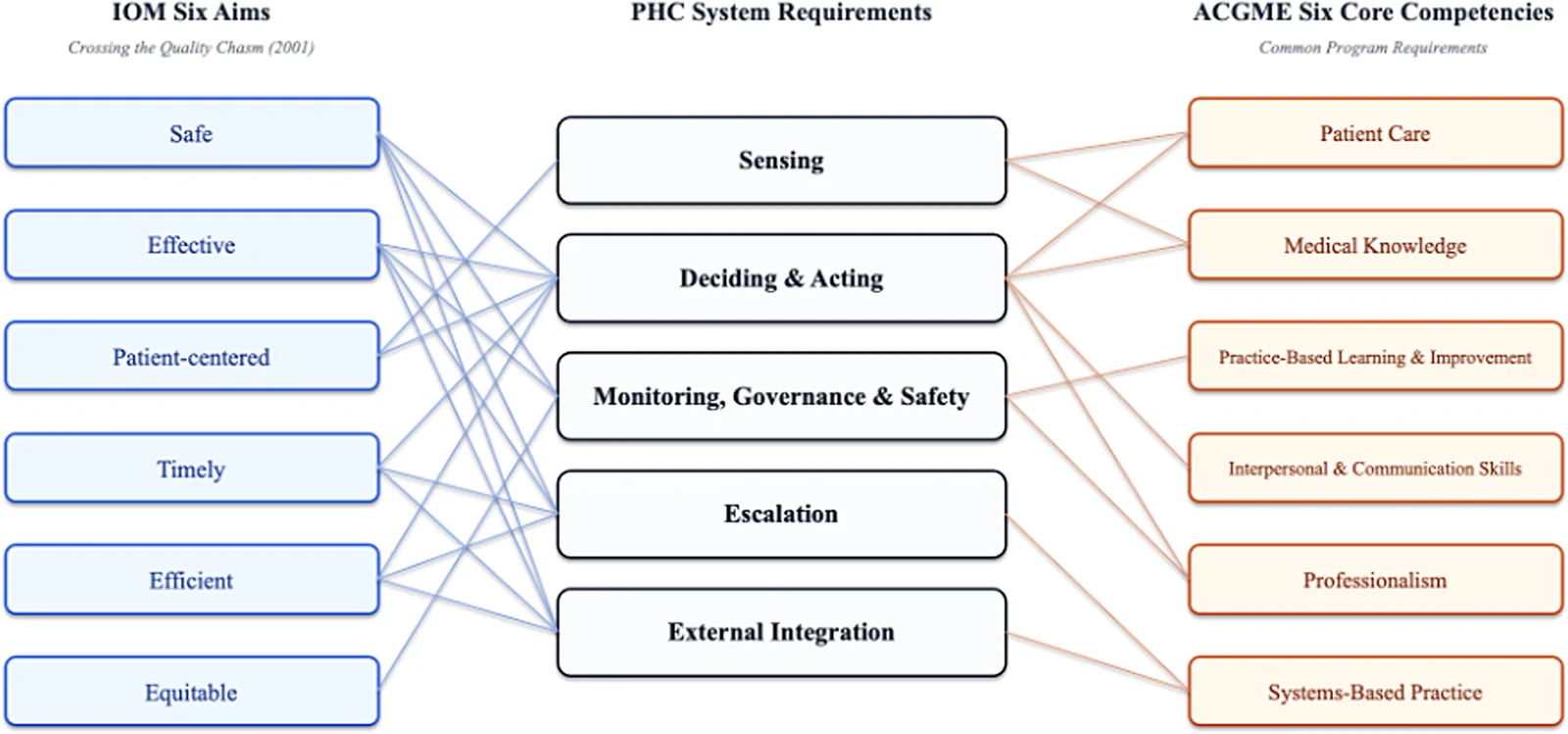
A crosswalk between the Personal Health Care requirements capabilities set and the IOM Six Aims and the ACGME Six Core Competencies.

## Conclusion

### Next Steps

The impact of digital health has yet to match the hype. Telemedicine and remote patient monitoring tools are important yet represent a small fraction of US healthcare [32]. Envisioning a functional Personal Health Care system became conceivable after the widely covered launch of ChatGPT on November 30, 2022 [33]. Suddenly, the varied Personal Health Care requirements went from hype and “out of reach” to “this can happen.”

Yet, plenty still needs to occur. Clinical validation for patient-facing AI is moving fast, though not ready for a system like this. CRAFT-MD introduced conversational evaluation over static examinations [34]. Google’s AMIE and the MIRA autonomous-agent study benchmarked AI against physicians on disease management and full clinical workflows [26, 35, 36]. Amazon’s PatientAgentBench went further still, evaluating patient-facing agents directly on safety and triage and clinical action execution, creeping closer to a validation standard for Personal Health Care [37]. ARPA-H’s ADVOCATE program (launched January 2026) is building an expanded validation approach, rigorous pre- and post-deployment testing with a supervisory AI overseer, for heart failure specifically [38].

On regulation, FDA cleared its first software as a medical device with a patient-facing conversational AI in June 2026 (UpDoc, for insulin management), but that clearance stopped at implementing clinician-specified plans, not autonomous decisions, which means responsibility when errors occur is still an evolving question [39]. In this *Digital Biomarkers* series on Personal Health Care, the authors take up the Personal Health Care system requirements above and put them under pressure.

### Sensing

An effective physician looks at a patient and can answer “what is going on?” Generative AI can only partially answer this today. It can interact with a patient and gather more history, but it cannot yet examine patients, nor can it effectively order, schedule, and interpret tests. That is coming with agentic AI applications. Patient-generated data from currently available wearables and monitors helps, but vital signs and activity tracking will only get the system so far. The right data, in the right context and setting, requires much more capable, specialty-grade sensors. Kathryn Mangold, Masoomeh Aslahishahri, and Zachi Attia from The Mayo Clinic, focused on cardiology, take up what specialty-specific technologies beyond today’s wearables will bring about the next level of specialty-relevant Personal Health Care.

### Deciding and Acting

Even with perfect sensing, the system must know what to do with what it sees, and the user has to know what to do with what the system tells them. Recent diagnostic-reasoning systems like Google’s AMIE and Microsoft’s MAI-DxO show the underlying inference is reaching clinical utility, but the gap between an AI’s recommendation and a patient’s safe action is closed by design choices: defaults, confidence display, escalation cues, and the orchestration of follow-on steps. Jesse Spooner and Neema Navai from The University of Texas MD Anderson Cancer Center test that idea in oncology, moving early testing into the home while keeping a clinician in charge of what happens next.

### Monitoring, Governance, and Safety

AI systems drift. Sensor calibrations slip. Populations change. A Personal Health Care system that does not monitor and re-validate itself becomes less safe and effective over time. And the legal architecture for inference-based, patient-facing systems is incomplete: HIPAA covers some of it, FDA covers some of it, state laws and the FTC fill some gaps, but the seams matter. Outcome tracking and learning, at both population and individual levels, sits inside this bucket. Michael Celone from the Department of Justice takes up data governance and liability for inference-rich personal health care systems.

### Escalation

Personal Health Care cannot tackle every problem. The system must know when to step back and route to a clinician, an urgent care visit, or the emergency department, and it must do so with enough context that the clinical handoff is fast. Hashem Zikry from Counsel Health maps the routing of higher-risk scenarios from home to clinic and beyond.

### External Integration

Personal Health Care will not exist in isolation. It will sit alongside, sometimes inside, the traditional health system. The question is whether it competes with that system, completes it, or both. James Whitbread and Peter Najjar from Johns Hopkins Medicine take up their hospital’s own AI camera rollout in operating rooms as a case study and important guidance for Personal Health Care on when these tools strengthen the health system and when they work against it.

If the Chicago Columbian Exposition of 1893 were held today, what would be highlighted? And 10 years from now? Gunn’s *Domestic Medicine* ran through 234 editions over 90 years because patients without doctors needed it. Harrison’s *Principles of Internal Medicine* has gone through 22 editions in 75 years because it serves a smaller audience of physicians [40]. Patients today face Gunn’s access problem but do not have his solution: a comprehensive, patient-facing, evidence-grounded reference designed for use without a doctor in the room. A better Harrison’s for doctors or a better Gunn’s for patients is not the answer.

The next Gunn’s is not a book. It is an AI-enabled Personal Health Care system.

To see still takes time, and as the Personal Health Care system acquires more ability to see the patient in the right context, it is finally within reach.

For the builders, investors, and clinical leaders: the trains have done what they can do. It is time for us to build the cars.

## Acknowledgments

The author is grateful to *Digital Biomarkers* and its editorial team for the opportunity to guest edit this series on Personal Health Care, and for their and the other authors’ support in bringing these contributions together. He is also incredibly grateful to the band of authors contributing to this Personal Health Care series, and for what they are doing to improve patient health and care.

## Conflict of Interest Statement

The author serves as an advisor to Sevaro Health and is part of the independent verification and validation JHU APL team supporting the ARPA-H ADVOCATE program.

## Funding Sources

This study was not supported by any sponsor or funder.

## Author Contributions

This work was solely conceived and executed by the author.
